# Enhancing the Efficacy of Melanoma Treatment: The In Vitro Chemosensitising Impact of *Vipera ammodytes* Venom on Human Melanoma Cell Lines

**DOI:** 10.3390/toxins17040152

**Published:** 2025-03-21

**Authors:** Giovanni Paolino, Matteo Riccardo Di Nicola, Carla Raggi, Serena Camerini, Marialuisa Casella, Luca Pasquini, Cristiana Zanetti, Vincenzo Russo, Santo Raffaele Mercuri, Luana Lugini, Cristina Federici

**Affiliations:** 1 Unit of Dermatology and Cosmetology, IRCCS San Raffaele Hospital, Via Olgettina 60, 20132 Milan, Italy; paolino.giovanni@hsr.it (G.P.); mercuri.santoraffaele@hsr.it (S.R.M.); 2 Faculty of Medicine and Surgery, Università Vita-Salute San Raffaele, 20132 Milan, Italy; 3 Istituto Zooprofilattico Sperimentale del Piemonte, Liguria e Valle d’Aosta, Via Bologna 148, 10154 Turin, Italy; 4 Department of Oncology and Molecular Medicine, Istituto Superiore di Sanità, 00161 Rome, Italy; carla.raggi@iss.it (C.R.); cristiana.zanetti@iss.it (C.Z.); luana.lugini@iss.it (L.L.); 5 Core Facilities, Istituto Superiore di Sanità, 00161 Rome, Italy; serena.camerini@iss.it (S.C.); marialuisa.casella@iss.it (M.C.); luca.pasquini@iss.it (L.P.); 6 Unit of Immuno-Biotherapy of Melanoma and Solid Tumors, IRCCS San Raffaele Hospital, Via Olgettina 60, 20132 Milan, Italy; russo.vincenzo@hsr.it

**Keywords:** antitumor peptides, chemoresistance, cisplatin, cytotoxicity, nose-horned viper, metalloproteinases, metastasis, snake venom, therapeutic adjuvants, viper

## Abstract

Research on viper venom has expanded into diverse medical applications, including cancer treatment. This study investigates the potential of *Vipera ammodytes* venom in oncology, evaluating its cytotoxicity and chemosensitising effects on malignant melanoma cells. Proteomic analysis identified 125 proteins in the venom, with Phospholipases A2, C-type lectins, and metalloproteinases among the most abundant components. These proteins are associated with cytotoxic, anti-proliferative, and tumor-inhibiting properties. Three melanoma cell lines (M001, Me501, and A375) were used to assess venom cytotoxicity. The IC50 values demonstrated consistent venom sensitivity across cell lines (approximately 1.1 µg/mL). Combined treatment with venom and cisplatin significantly increased the cytotoxicity compared to single-agent treatments. Notably, venom enhanced the sensitivity of cisplatin in resistant cell lines (M001 and Me501), increasing cell mortality by up to 40%. The A375 cell line, inherently more sensitive to cisplatin, exhibited additional cytotoxic effects only at higher venom doses. The morphological changes observed under microscopy confirmed venom-induced cellular changes, further supporting its potential as an anti-cancer agent. The selective targeting of melanoma cells by venom components, particularly in muscle-associated metastases, suggests a unique therapeutic niche. While cisplatin was chosen for this pilot study due to its established cytotoxicity, future research will explore venom combinations with contemporary treatments such as immunotherapy and targeted therapies. Although preliminary, these findings provide a foundation for integrating venom-based strategies into advanced melanoma protocols, aiming to improve outcomes in resistant or metastatic cases.

## 1. Introduction

Research on viper venom covers a variety of fields and applications, aimed at improving medical treatments and better understanding the venom’s biochemical properties. The main fields involving viper venom research include the development of universal antivenoms, studying venom composition to better understand geographic and interspecific proteomic variations, and developing more efficient antivenoms and biomedical applications [1,2,3]. Indeed, in recent years, viper venom has been involved in drug development, as well as in cosmetics [4]. Following the discovery of an anti-hypertensive drug (Captopril) derived from the venom of the snake *Bothrops jararaca* (Wied-Neuwied, 1824) in 1975, various venom-based drugs with different medical applications have been developed [1,5,6].

Indeed, snake venom toxins show several potential therapeutic activities against cancer due to their ability to target and disrupt key cellular pathways with a high level of selectivity. Several toxin families (such as phospholipases A2 [PLA2s], L-amino acid oxidases [LAAOs], three-finger toxins [3FTxs], metalloproteases [MPs], disintegrins, and C-type lectins) exhibit cytotoxic, pro-apoptotic, and antiangiogenic properties [7,8]. One of the main mechanisms by which snake venom toxins exert their anticancer effects is the induction of apoptosis via both intrinsic and extrinsic pathways. Regarding the intrinsic pathway, PLA2s, LAAOs, and 3FTxs can induce membrane disruption, triggering the release of cytochrome C from mitochondria into the cytoplasm and activating the caspase cascades involved in programmed cell death. Contrariwise, the extrinsic apoptotic pathway is driven by ligand–receptor interactions that further enhance cellular demise. LAAOs also contribute to tumor cell death by catalyzing the oxidative deamination of L-amino acids, leading to the production of reactive oxygen species (ROS). The accumulation of ROS results in oxidative stress, membrane damage, and apoptosis, while its regulation is influenced by catalase activity. Beyond direct cytotoxic effects, snake venom components interfere with the essential signaling pathways that regulate tumor growth and metastasis. Disintegrins (DIS) play a pivotal role in inhibiting integrin-mediated focal adhesion kinase (FAK) signaling, altering cell adhesion, migration, and angiogenesis, therefore preventing tumor cells from establishing the vascular networks necessary for sustained growth and dissemination. At the same time, PLA2s have been shown to bind vascular endothelial growth factor receptors (VEGFRs), thereby reducing the vascularization, invasion, and metastatic potential of tumors [7,8].

The medical fields, excluding cosmetics, in which snake venoms have been used range from coagulation disorders (metalloproteinases and phospholipases are used to develop drugs that control blood clotting), pain disorders, antiparasitic and antimicrobial agents (e.g., oxidase amino acid detected in *B*. *jararaca*) to cardiovascular treatments that address issues such as thrombosis (e.g., botroxobin is present in snakes of the genus *Bothrops*) [2,3]. In this context, another important medical area in which snake venom has been used is cancer treatments. Specifically, snake venoms composed of about 90–95% of the dry weight of proteins and peptides, which are responsible for the main biological effects, have been studied for their potential anti-tumoral activity, which includes inhibiting tumor growth and progression, and inducing apoptosis in malignant cells [9]. Snake venom, containing key biologically active factors, can be classified into several groups based on the effect induced, such as cytotoxic, neurotoxic, cardiotoxic, and hemotoxic effects [9]. At the same time, the same venom can exhibit different types of toxicity. To perform these toxic actions, the venom is biochemically composed of peptides, enzymes, proteins, chemicals, inorganic cations (e.g., sodium, zinc, calcium, magnesium, potassium), carbohydrates, free amino acids, and lipids [9,10]. These components may have a specific effect on tissues, systems and apparatus, and also exhibit effects on several types of cancer, including breast cancer, lung cancer, prostate cancer, colon cancer, leukemia, glioblastoma and melanoma [7,8,11,12,13].

Melanoma is a type of skin cancer that originates from melanocytes; its incidence has been rising globally, with an estimated 106.000 new cases per year in the USA and 144.000 new cases per year in Europe [14]. Surgical excision is often the first line of treatment for localised melanomas, while systemic treatments are needed in the case of disseminated disease. The average five-year survival rate for melanoma varies widely depending on its stage at diagnosis: around 99% for localised melanoma, 66% for melanoma with regional spread, and 27% for metastatic melanoma [15]. In recent years, important therapeutic advances have been made in the treatment of advanced melanoma, as well as in adjuvant settings. Targeted therapies (e.g., BRAF/MEK inhibitors) are used for melanoma with BRAF mutation; meanwhile, immunotherapies enhance the body’s immune response against cancer cells, with ipilimumab blocking CTLA-4, nivolumab and pembrolizumab as PD-1 checkpoint inhibitors, and relatlimab being used as a LAG-3 checkpoint inhibitor [16]. Unfortunately, approximately 40–50% of patients develop resistance to targeted therapies, while around 20–30% of patients initially respond to immunotherapy; however, many develop resistance over time [16,17]. The mechanisms of resistance include secondary mutations, the activation of alternative signalling pathways, phenotypic plasticity, changes in the tumor microenvironment, the expression of inhibitory molecules, and the loss of tumor antigens [16]. These challenges highlight the need for ongoing research into more effective treatments. In this context, new potential therapies or therapeutic adjuvants could play an important role in the management of this malignancy [17]. Researchers have begun to highlight the cytotoxic potential of venoms in general and snake venom in particular. Indeed, many of the proteins and peptides in snake venom have not yet been thoroughly investigated, and exploring this cocktail of components could represent a new therapeutic approach to cancer. In this study, we performed experiments to characterise the proteic profile and efficacy of melanoma treatment, as well as the chemosensitizing impact of the Nose-horned viper’s venom on human melanoma cell lines [16,17].

The Nose-horned viper, *Vipera ammodytes* (Linnaeus 1758), is a species complex (see [18,19]) distributed from northeastern Italy and southern Austria across the Balkans to the Ionian and Aegean Islands, extending into Asia Minor [20,21,22]. It is considered the most medically significant and one of the most venomous snakes in Europe [19,23,24,25].

The venom of *V. ammodytes* was chosen over other European vipers due to its remarkable composition, which includes a high abundance of PLA_2_, CTL, and SVMP, which have been documented for their cytotoxic and anti-proliferative properties (e.g., [1,25,26,27]). *Vipera ammodytes* venom is particularly promising for oncological applications, including its ability to sensitise melanoma cells to chemotherapeutic treatments.

## 2. Results

### 2.1. Viper ammodytes ammodytes Venom Proteins Detected

**Figure 1:**
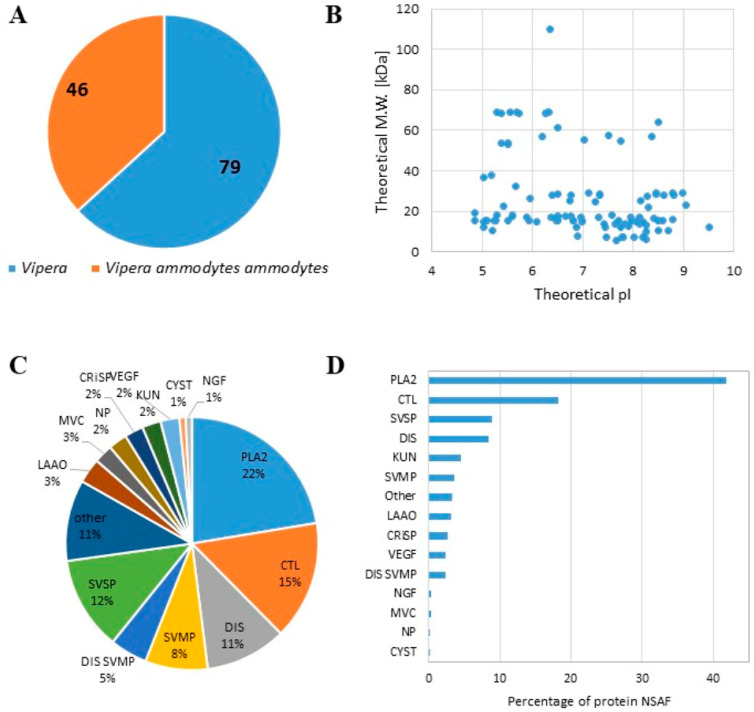
Proteomic analysis of the lyophilised venom of Balkan *Vipera ammodytes ammodytes*. (**A**) Pie chart showing the number of proteins identified as belonging to *Vipera ammodytes ammodytes* or other *Vipera* species. (**B**) Scatter plot showing the theoretical isoelectric point (pI) and molecular weight (M.W.) of the detected proteins. (**C**) Pie chart showing the distribution of the detected proteins (the percentage is reported in each slice) related to each family. (**D**) Bar graph with the number of protein NSAFs detected for each family (PLA2; SVSP; SVMP; LAAO: l-amino acid oxidase; KUN: kunitz peptide; CRiSP: cysteine-rich secretory protein; MVC: minor venom component; CTL; DIS; NP: natriuretic peptide; CYST: cystatin; VEGF: vascular endothelial growth factor; MVC: minor venom component).

We characterized the proteome content of the lyophilised venom of Balkan *Vipera ammodytes ammodytes* (L1117, Latoxan Laboratory, Valence, France). Two different protocols for proteomic analysis were applied: proteins were denatured at 95 °C and directly digested by trypsin or were primarily precipitated and then enzymatically digested. We combined the data obtained using both procedures and identified a total of 125 proteins, (Table S1), 46 of which had specifically matching proteins present in *V. ammodytes ammodytes* (Figure 1). A similar number of proteins (more than 100) was detected in both the precipitated and directly digested samples, with over 80% of proteins identified by at least two peptides in both protocols across all replicates (Figure S1). Most of the identified proteins had a theoretical molecular weight under 40 kDa, and their theoretical pI ranged between 4.5 and 9.5 (Figure 1B). We classified the identified proteins based on their affiliation with one of 12 families, following Tasoulis and Isbister [28]. We observed an increase in the phospholipase A2 (PLA2) family (22%) in terms of both the number of protein species (Figure 1C) and the amount expressed in NSAF (Figure 1D). Successively, C-type lectin (CTL) 15%, disintegrin (DIS) 11%, snake venom metalloprotease (SVMP) 8%, and serine protease (SVSP) 12% were the more represented families (Figure 1C,D). These results are consistent with proteomics studies performed on the same viper species [25,26].

### 2.2. IC50 Cisplatin and Viper Venom Values of Melanoma Cell Line

**Figure 2:**
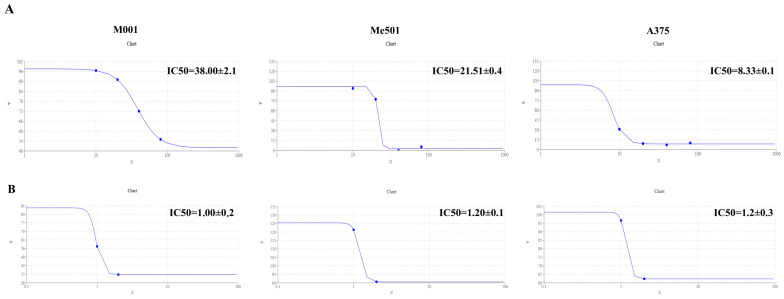
IC50 calculation. (**A**) Dose–response curve of M001, Me501 and A375 cells cultured in 24-well plates and treated with increasing concentrations of cisplatin (0–80 μM) for 24 h. The y-axis shows the rate of cell viability, while the x-axis shows the cisplatin concentration (μM). The dose–response curve was obtained by using https://www.aatbio.com/tools/ic50-calculator (accessed on 25 January 2025). The graph is representative of three experiments. (**B**) The dose–response curve of M001, Me501 and A375 cells cultured in 24-well plates and treated with decreasing concentrations (0–2.5 μg/mL) of viper venom for 24 h. The y-axis shows the rate of cell viability, and the x-axis shows the viper venom concentration (μg/mL). All experiments were performed in triplicate and repeated at least three times. Data are expressed as mean ± SD.

Firstly, we verified the lack of toxicity that viper venom poses to human HUVEC cells. Subsequently, we evaluated the IC50 of cisplatin and viper venom on M001, Me501, and A375 melanoma cell lines. The results demonstrated that the M001 was the most cisplatin-resistant cell line (IC50 = 38 µM), compared to Me501 (IC50 = 22.85 µM). A375 was the most sensitive cisplatin cell line (IC50 = 8.33 µM) (Figure 2A). However, the sensitivity of viper venom was similar across all three melanoma cell lines, with an IC50 of about 1.1 µg/mL (Figure 2B).

### 2.3. Cytotoxic Effect of Combined Treatment on Melanoma Cell Lines

**Figure 3:**
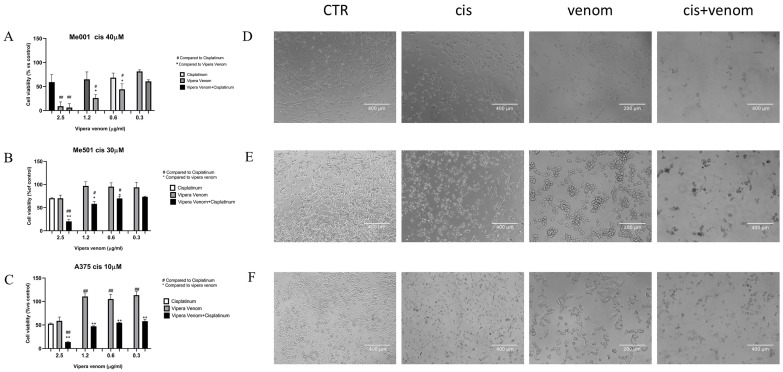
Effect of viper venom alone and combined with cisplatin in M001 (**A**), Me501 (**B**) and A375 (**C**) melanoma cells for 24 h of treatment. The cell viabilities were determined after the final treatment. Each point represents the mean ± SD relative to the control cells of three independent experiences, with at least five images for each point being analysed, *n* = 5. *^,#^ Statistically significant vs. viper venom at *p* < 0.05; **^,##^ Statistically significant vs. viper venom at *p* < 0.01; Statistical analyses were performed by using a one-way ANOVA test. (**D**–**F**) morphological analysis of changes in phase contrast microscopy of M001, Me501 and A375 cell lines, respectively. CTR: control, Cis: cisplatin treatment, venom: viper venom treatment, cis + venom: combined treatment.

After the IC50 calculation, we performed combined drug treatment, in which cisplatin was used at a fixed dose close to the IC50 calculated for each cell line: 40µM for M001, 30 µM for Me501, and 10 µM for A375 (three cell lines with a clear and different degree of cisplatin resistance). All cell lines were treated simultaneously with increasing doses of viper venom for 24 h: 2.5, 1.2, 0.6 and 0.3 µg/mL. The FACS analysis of cytotoxicity showed that for the combined treatment, the rate of cell mortality significantly increased compared to the single treatments (Figure 3). In the cell lines resistant to cisplatin (M001, IC50 = 40 µM; Me501, IC50 = 30 µM), the use of viper venom increased chemosensitivity. In fact, in M001, the cell mortality increased by about 20 and 40% at the lowest doses of 0.3 and 0.6 µg/mL viper venom (Figure 3A), and in Me501, it increased by about 30% with the 1.2 µg/mL dose of viper venom (Figure 3B). The A375 cells are inherently more sensitive to cisplatin alone. In these cells, the combined treatment showed a significant reduction in viability (40%) at the highest dose of 2.5 µg/mL viper venom (Figure 3C).

The images obtained via phase-contrast microscopy show the morphological changes in the cells, particularly following venom treatment, where the cells mostly became spherical and agglomerated while maintaining viability. Massive cellular damage and mortality were evident in the combined treatment.

## 3. Discussion

Melanoma develops due to the damage caused to melanocytes by excessive exposure to UV light or radiation. This damage induces DNA mutations that promote cancerous changes, leading to uncontrolled cell proliferation and tumor formation. When detected early, melanoma can often be effectively treated with surgery, significantly improving survival rates [29]. However, treatment becomes more challenging once the cancer metastasises to distant organs. Immunotherapy (e.g., ipilimumab, nivolumab, pembrolizumab) and target therapy (e.g., vemurafenib/cobimetinib, dabrafenib/trametinib in case of BRAF mutation) have increased the progression-free and overall survival in melanoma patients [29,30]. Unfortunately, despite this progress, melanoma remains a solid tumor with one of the highest mutational loads, also having the capacity to develop resistance to systemic treatments in 20–50% of cases [30]; this results in a 5-year survival rate of only 27% for metastatic disease [29,31]. Therefore, continual progress in research in this area is essential for developing increasingly effective therapies. Specifically, novel ideas and pharmaceutical strategies that reduce these resistance phenomena will play an increasingly important role in the genesis of new therapeutic strategies and combinations, aiming to enhance the efficacy of primary treatments.

To date, proteins, peptides, and alkaloids extracted from different venoms have demonstrated therapeutic efficacy in the treatment of cancer [1,8,9,32,33,34,35,36,37,38,39,40,41,42,43,44,45]. Specifically, recent studies have found that snake venom can induce death in malignant melanoma cells, exhibiting anti-proliferative activity [46,47,48]. In particular, snake venoms can modulate or antagonise various cellular physiological functions, making them valuable in pharmacological research for discovering bioactive molecules that target tumor receptors [46].

The venom of *Vipera ammodytes* primarily comprises PLA2, VEGF, SVSP, SVMP, LAAO, CRISP, and CTL [49]. Consequently, each of these components may play a role in improving cancer treatment [49,50]. To deepen our knowledge of the therapeutic potential of *V. ammodytes* venom, we characterised its proteic profile and efficacy in melanoma treatment, and for the first time, evaluated its relative chemosensitising impact on human melanoma cell lines. To assess the improvement in the efficacy of melanoma treatment using viper venom, we employed cisplatin as a reference drug. While acknowledging that cisplatin is an older chemotherapeutic agent, it was chosen in this pilot study due to its well-established cytotoxic activity against melanoma cells.,

We biochemically characterised the venom protein composition of Balkan *V. ammodytes*, and the results showed that of the 125 proteins identified, 46 specifically matched the proteins present in the *V. ammodytes ammodytes* database. We found that the PLA2, CTL, DIS, SVMP, SP families were enriched. Our data align with previous work and broaden our knowledge regarding the composition and efficacy of venom as an antitumor drug [25,51,52]. Interestingly, the *V. ammodytes* venom IC50 in the three studied melanoma cell lines with different levels of resistance to cisplatin was similar (about 1.1 µg/mL), likely due to the nature of the ‘drug’, which is proteic for venom and chemical for cisplatin. The mechanisms of resistance that a cell develops towards a chemical compound/drug differ from those that develop towards a proteic mix of drugs [53,54], which supports the use of *V. ammodytes* venom as a potential proteic anti-cancer drug.

Moreover, we evaluated the effect of combined venom and cisplatin treatment on melanoma cells with different degrees of cisplatin resistance. In the cell lines resistant to cisplatin (M001, IC50 = 40 µM and Me501, IC50 = 30 µM), the use of viper venom made them more chemosensitive compared to the more cisplatin-sensitive A375 cell line. Intriguingly, the M001 melanoma cell line, originating from muscular metastases, showed the best response to treatment with *V*. *ammodytes* venom alone and in combination with cisplatin.

Considering that the metastasis of melanoma to skeletal muscle is often associated with a poor prognosis and is frequently not responsive to treatments, this therapeutic response could provide opportunities for future treatments [55]. Indeed, the myotoxic effects of snake (Viperidae family) venom are mostly due to non-enzymatic toxins and enzymes of the PLA2 type, which target myocytes, cause the destruction of striated muscle cells known as rhabdomyolysis, and result in minor or even major myonecrosis [56,57]. Therefore, this selectivity for myocytes could be useful in adjuvant treatments.

A limitation of this study is the use of cisplatin instead of immunotherapy or targeted therapies, which are the current standards for melanoma treatment. However, cisplatin was chosen due to its well-characterised cytotoxic mechanism and extensive preclinical data, which provide a solid foundation for evaluating the feasibility of this combination.

Several studies have focused on screening the components of viper venom for use as templates for designing anticancer molecules [58,59,60,61]. In this study, we propose the utilisation of venom due to its ability to enhance the sensitivity of cancer cells to chemotherapy, a treatment to which they are resistant to varying degrees. Our findings demonstrate that at the proposed venom concentrations, no toxicity is observed; however, toxicity emerges only in combination with the chemotherapeutic agent. These findings provide substantial evidence supporting our hypothesis that the venom effectively targets and modifies altered pathways in tumor cells, thereby overcoming the chemoresistance of tumors [62,63]. A more thorough analysis would be necessary in the future to elucidate the potential of the venom in toto and its individual components as possible chemosensitising anticancer drug candidates.

## 4. Conclusions

This study provides preliminary evidence supporting the potential therapeutic value of combining snake venom, a protein mix compound, with melanoma treatments. The results highlight the enhanced cytotoxicity achieved through this combination, suggesting that snake venom may improve the efficacy of existing therapeutic strategies, possibly overcoming melanoma resistance.

## 5. Materials and Methods

### 5.1. Melanoma Cell Lines and Chemical Reagents

The human metastatic melanoma cell lines used were as follows: (i) Me501, from a subcutaneous metastatic tumor lesion [64]; (ii) M3M001, from a muscular metastases (referred to as M001), provided by one of the authors (V.R.) [65]; and (iii) A375, an in situ skin malignant melanoma (American Type Culture Collection, Manassas, VA, USA). The melanoma cells were cultured in RPMI 1640 medium supplemented with 10% fetal bovine serum, 100 IU/mL penicillin, 100 μg/mL streptomycin (Life Technologies, Gaithersburg, MD, USA), and 2 mmol/L glutamine (Life Technologies) in a 5% CO_2_ environment at 37 °C.

All cell lines were negative for mycoplasma contamination, as routinely tested by a PCR Mycoplasma detection kit (Venor GeM; Minerva Biolabs, Berlin, Germany).

The lyophilised venom of Balkan *Vipera ammodytes ammodytes* (Latoxan, Valence, France, code L117) was diluted in 1 mL of 1 M ammonium bicarbonate [66]. Cisplatin powder (PHR1624 Sigma Aldrich, St. Louis, MO, USA) was resuspended in 0.5% of dimethyl sulfoxide (DMSO). The values indicated are already subtracted from the value of the control with DMSO alone.

### 5.2. Melanoma Cell Lines and Chemical Reagen Proteomic Analysis

Protein sample preparation for mass spectrometry analysis was carried out following two protocols. In the first protocol, snake venom proteins (Latoxan laboratory, Valence, France) were dissolved in 100 mM of ammonium bicarbonate (AMBIC), subjected to cysteine reduction by 25 mM of tris (2-carboxyethyl) phosphine (TCEP, Sigma Aldrich, MO, USA) for 10 min and then subjected to alkylation by 10 mM of IAM (Sigma Aldrich) for 30 min in the dark. The proteins were then precipitated by incubation with six volumes of an organic solution (methanol, acetone and ethanol; 25%, 25%, and 50% *v*/*v*, Sigma Aldrich) at −20 °C overnight. The protein pellet was centrifuged at 4 °C, suspended in 1 M urea (Sigma) and 25 mM of ammonium bicarbonate (AMBIC), and digested overnight with trypsin (Promega Corporation, Madison, WI, USA) at a substrate-to-enzyme ratio of 50:1 (*w*/*w*) at 37 °C on a mixer heat block. In the second protocol, snake venom proteins were denatured for 10 min at 95 °C in 25 mM of AMBIC and, without a protein precipitation step, directly digested after cysteine reduction and alkylation under the previously described conditions. The peptide mixture derived from each protocol was injected four times into an Ultimate 3000 UHPLC (Thermo Fisher Scientific, San Jose, CA, USA) coupled with an Orbitrap Fusion Tribrid mass spectrometer (Thermo Fisher Scientific, CA, USA). The peptides were desalted on a trap column (Acclaim PepMap 100 C18, Thermo Fisher Scientific) and then separated on a 45 cm long silica capillary (ICT 36007508-50-5, MS WIL, Aarle-Rixtel, The Netherlands). The analytical column was encased by a column oven (Sonation; 40 °C during data acquisition) and attached to a nanospray flex ion source (Thermo). The peptides were separated by a 120 min long gradient of buffer A (95% water, 5% acetonitrile, and 0.1% formic acid) and buffer B (95% acetonitrile, 5% water, and 0.1% formic acid) at a flow rate of 250 nL/min. The mass spectrometer was operated in positive ion mode, using a data-dependent acquisition strategy. Precursor ion scanning was performed using Orbitrap in the 350–1550 *m*/*z* range with a 120 K resolution. Data-dependent acquisition was performed in top-speed mode (3 s long maximum cycle time): the most intense precursors were selected using a monoisotopic precursor selection (MIPS) filter with charge >1, with a quadrupole isolated and fragmented by higher-energy collisional dissociation (HCD) (30%). The product ion spectra were acquired in the ion trap with a rapid scan rate.

### 5.3. Data Processing

Peptide spectra were searched using Proteome Discoverer 2.4 (Thermo Fisher Scientific), with Sequest HT used as the search engine against two databases downloaded from UniProtKB/Swiss-Prot: *Vipera ammodytes ammodytes* (Rev_Unrev) database (Release 2024; 126 sequences) and the *Vipera* (Rev_Unrev) database (Release 2024; 1559 sequences). All mass spectrometry data have been deposited in the ProteomeXchange Consortium via the MASSIVE repository with the dataset identifier MSV000096836. Spectral matches were filtered using the Percolator node, with a 1% q-value-based false discovery rate (FDR). Only master proteins were considered, and only specific trypsin cleavages with two miscleavages were admitted. Cysteine carbamidomethylation was set as the static modification, while methionine oxidation and N-acetylation at the protein terminus were set as variable modifications. The precursor and fragment mass tolerance were set to 15 ppm and 0.6 Da, respectively. To evaluate the protein relative abundance, we calculated the Normalized Spectral Abundance Factors (NSAFs) [67]. For each protein, the SpC/L value was calculated by dividing the number of spectral counts (SpC) able to identify the protein by its length (L). Then, the NSAFs were obtained by normalising each SpC/L to the sum of the SpC/L of all the proteins identified in the mixture. All mass spectrometry proteomics data have been deposited in the ProteomeXchange Consortium via the PRIDE/MASSIVE repository under the dataset MSV000096836.

### 5.4. IC50 Evaluation

The half-maximal inhibitory concentration (IC50) values for viper venom and cisplatin in the M001, Me501 and A375 melanoma cell lines were determined at the following concentrations: 2.5, 1.2, 0.6, 0.3 µg/mL for viper venom and 80, 40, 20 and 10 µM for cisplatin, respectively; this was performed using the IC50 calculator available at https://www.aatbio.com/tools/ic50-calculator (accessed on 25 January 2025). All experiments were performed in triplicate, and the SD was calculated.

### 5.5. Cytotoxic Assay

Melanoma cells were plated at 5 × 10^4^ cells per well in buffered RPMI medium. The cells were treated with venom and cisplatin alone or in combination at different doses after 24 h. The M001, Me501 and A375 cell lines were co-treated with 2.5, 1.2, 0.6 and 0.3 µg/mL of viper venom and fixed doses of cisplatin at 40, 20 and 5 µM, respectively. Cell death was evaluated by staining the cells with propidium iodide (PI) following the manufacturer’s instructions (BioVision Incorporated, Milpitas, CA, USA). After staining for 15 min, cells were acquired using a Cytoflex S cytometer (Beckman Coulter, Inc., Brea, CA, USA), and the data were subsequently analysed with CytExpert software, version 2.5.0.77 (Beckman Coulter, Inc., USA). All experiments were performed in triplicate and repeated at least three times.

### 5.6. Microscopy

After 24 h treatment, the cell morphology was evaluated using phase contrast microscopy. Five images for each experiment, which were repeated 3 times, were acquired on live cells by using an EVOS-FL microscope (Life Technologies).

### 5.7. Statistical Analysis

Data are presented as means ± SD, with n representing at least three independent sets of experiments and triplicate wells per experiment. Statistical analyses were performed using GraphPad Prism Software 8.02 (GraphPad, La Jolla, CA, USA). A one-way ANOVA was performed to compare the means among multiple groups, followed by a post hoc test. Values were considered statistically significant if the probability was below the 5% confidence level (*p* < 0.05).

## Data Availability

The original contributions presented in this study are included in the article/Supplementary Material. Further inquiries can be directed to the corresponding authors.

## Supplementary Materials

The following supporting information can be downloaded at: https://www.mdpi.com/article/10.3390/toxins17040152/s1, Table S1: List of venom proteins; Figure S1: Number of proteins identified with either one or at least two peptides in each sample that was either directly digested or precipitated before enzymatic digestion.
