# Supplementary material for: Enhancing the Efficacy of Melanoma Treatment: The In Vitro Chemosensitising Impact of Vipera ammodytes Venom on Human Melanoma Cell Lines
Source: Toxins (Basel). 2025 Mar 21;17(4):152. doi: 10.3390/toxins17040152 (PMC12031473; doi:10.3390/toxins17040152)
Supplement: Supplementary file 1 [file toxins-17-00152-s001.zip › Supplementary Figure S1.pdf]

Supplementary Materials

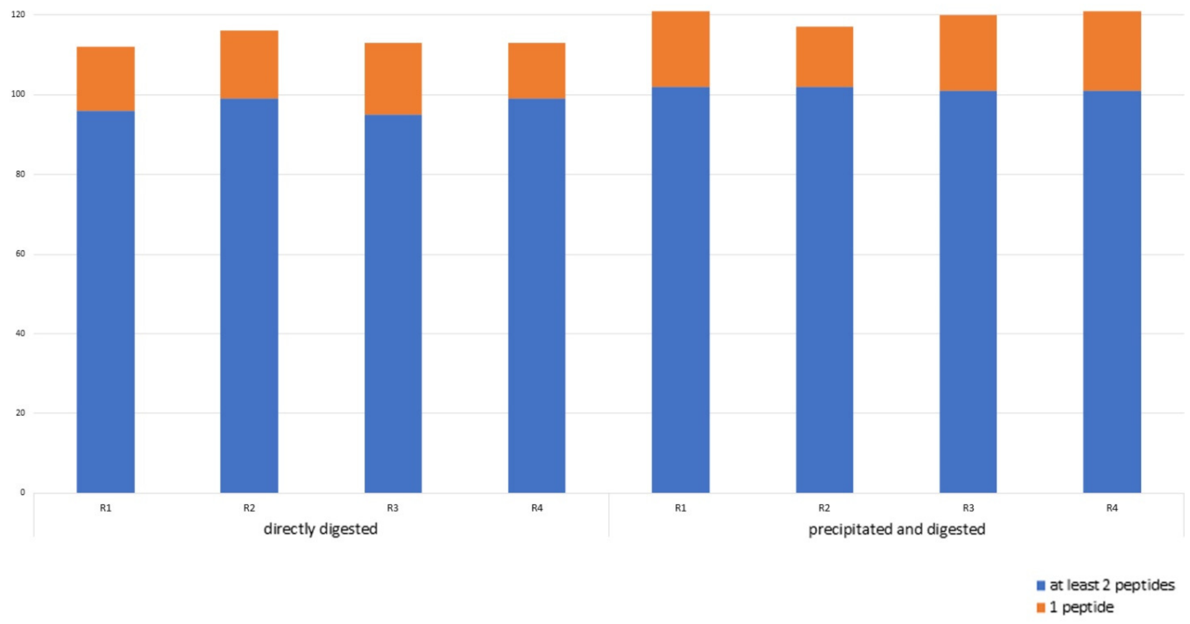

Figure S1. Number of proteins identified with either one or at least two peptides in each sample that was either directly digested or precipitated before enzymatic digestion.
